# The concept of virginity from the perspective of Iranian adolescents: a qualitative study

**DOI:** 10.1186/s12889-020-08873-5

**Published:** 2020-05-19

**Authors:** Mohammad Hossein Mehrolhassani, Vahid Yazdi-Feyzabadi, Saeid Mirzaei, Farzaneh Zolala, Ali-Akbar Haghdoost, Nadia Oroomiei

**Affiliations:** 1 Health Services Management, Medical Informatics Research Center, Institute for Futures Studies in Health, Kerman University of Medical Sciences, Kerman, Iran; 2 Health Policy, Health Services Management Research Center, Institute for Futures Studies in Health, Kerman University of Medical Sciences, Kerman, Iran; 3 Health Policy, Department of Health Management, Policy and Economics, School of Public Health, Bam University of Medical Sciences, Bam, Iran; 4 Epidemiology, Social Determinants of Health Research Center, Institute for Futures Studies in Health, Kerman University of Medical Sciences, Kerman, Iran; 5 Epidemiology, Health Modeling Research Center, Institute for Futures Studies in Health, Kerman University of Medical Sciences, Kerman, Iran; 6 Bam University of Medical Sciences, Sardaran Shahid Square - Shahid Rajaei Boulevard, Bam, Postal Code: 7616913555 Iran

**Keywords:** Virginity, Adolescents, Iran, Virginity policy

## Abstract

**Background:**

Premarital sex can increase the risk of sexually transmitted diseases (STDs) in adolescents, and lack of premarital sex can be considered as a reliable policy for STD prevention, which is used by some countries such as Iran. Since the success of this policy is related to the concept of virginity, the present study was conducted to determine the concept of virginity from the perspective of Iranian adolescents.

**Methods:**

In this qualitative study with phenomenological approach, the research team visited public places, including parks and coffee shops, and interviewed a number of 15–19-year-old adolescents. The data were collected using in-depth interviews with semi-structured questions and analysed using thematic analysis method.

**Results:**

Several themes, including virginity as the lack of emotional relationship with the opposite sex, lack of physical contact, nonpenetrative relationship, virginity as a myth, virginity as a commitment, having an intact hymen, and not knowing the meaning of virginity, were extracted from the data.

**Conclusion:**

The most reliable policy on STD prevention is the lack of premarital sex. The success of this policy is related to the concept of virginity. The findings of this study showed that the participants did not consider physical contact as the breach of virginity. This may indicate that the policy of not having sex before marriage or lack of premarital sex is not enough and Iranian adolescents are at risk of STDs. Therefore, policymakers must take steps towards modifying the concept of virginity in the adolescents’ value system and provide and implement educational programs on sexual health for adolescents.

## Background

Sexually transmitted infections (STIs) such as HIV, chlamydia, and syphilis are among the main health problems in the world and have a high prevalence in the Middle East and North Africa (MENA) region [1]. The MENA region is the third fastest-growing HIV/AIDS epidemic in the world. Statistics show that HIV and STIs are more prevalent in 15–29 age group [2]. Accordingly, there are concerns about the widespread epidemics in the MENA countries and the risk of HIV/AIDS and STDs among adolescents [3]. Sexual health education is an effective prevention strategy that reduces the risk of STDs [4]. However, as premarital sexual behaviour is not accepted in many countries in this region and sexual health education is limited by economic, cultural, political, and religious factors [3], and there is limited access to sexual health education, little education is offered to single people about high-risk sexual behaviours. Hence, virginity as a tradition or a religious value can become a tool to prevent STIs [4, 5].

Virginity as the lack of sexual intercourse before marriage has been considered as a value and sign of piety in many religions for centuries [6]. Nevertheless, the cultural and social place of virginity and the attitude of people toward premarital sexual relationship has been changed considerably within the last few years following the incorrect perceptions of virginity and changes in the world value system [7]. Premarital sex is accepted in many countries and measures have been taken to secure this relationship [8]. On the other hand, in some countries, virginity is considered a value for females, especially at the time of marriage. It is considered as a sign of woman’s chastity and respectability [9]. For example, in Zimbabwe, a woman’s virginity is a sign of loyalty to marital life. Generally, different countries have different approaches to this subject, which is based on their cultural and religious context [10].

Iran is a MENA country with Islamic ideology where sexual relationships are accepted only within the marriage framework. Also, fathers’ permission is needed for females in their first marriage. In Iran, only a one-day mandatory training session, known as compulsory training, is held for newly engaged couples as premarriage education, and no other formal education on sexual behaviour is provided for young people [11]. In the context of Iran, sexual relation outside marriage is prohibited by law and religion, and it is associated with penalties and punishments [12]. For example, if a female with no previous marriage history gets married and hides her lack of virginity from her husband, the law allows the husband to divorce her [13, 14]. Therefore, girls’ virginity in the context of Iran is of great importance and lack of virginity is considered a stigma. Accordingly, any emotional relationship and contact between men and women outside marriage are unacceptable and the stigma of such relationship is much greater for women than for men [15]. Most of the women who break the taboo of premarital sex undergo hymenoplasty to increase their chances of successful marriage [16]. Adolescents are at a serious risk of premarital sex [17].

In Iran, adolescents’ (14%) behaviours affect their health and the health of next generations [18, 19]. Moreover, due to biological changes that occur naturally during adolescence period, the desire to establish a relationship with the opposite sex is intense [20]. Therefore, this kind of relationship may have many consequences, such as losing virginity, unwanted pregnancy, illegal abortion, transmission of AIDS and other STDs, illegal hymenoplasty, depression, leaving school, conflict between parents and their divorce, social exclusion, violence, and even honour killings [16].

Virginity as an abstinence strategy is the main strategy of Iranian policymakers to counter high-risk sexual behaviours of unmarried people. In fact, from the policymakers’ point of view, virginity means having no sex. As lack of virginity before marriage is not acceptable in the Iranian society, the policymakers assume that the prevalence of premarriage sexual relations is insignificant, and thus there is no considerable risk of STDs for single people [21].

Although keeping virginity claims to be the most reliable policy for STDs, the success of this policy is closely related to the meaning and definition of virginity [22, 23]. In Iran, this policy has been adopted because of the unacceptability of premarital sex in Iran, which is also reinforced by national laws. However, the following question is raised: Does virginity mean not having sex from Iranian adolescents’ perspective? Or does it have a different meaning from the Iranian adolescents’ perspective? In this case, the policymakers need to adopt other policies in addition to the abstinence policy to prevent STDs in adolescents. Thus, the aim of this study was to determine the concept of virginity and purity from the perspective of Iranian adolescents.

### Setting

This study was conducted in Kerman, the capital of Iran’s largest province, located in southeastern Iran. According to the Census of 2017, the population of Kerman was 632,162 and the population of 10–19-year-olds was 93,552 [24].

## Methods

### Study design

This was an exploratory qualitative study and the research team aimed to explore adolescents’ perceptions of virginity. Also, a phenomenological framework was used in this study. The study population included all adolescents living in Kerman. Participants were identified using convenience sampling method. Inclusion criteria were as follow: (1) being an adolescent and (2) willingness to participate in the study. Although schools were suitable sites for sampling, Iran’s Ministry of Education does not allow the conduction of studies on sex-related issues in schools. Therefore, due to the lack of cooperation of the Ministry of Education, the researcher attended sites where adolescents were more likely to be present. To select samples, the research team visited different public places such as parks, coffee shops, shopping malls, hookah houses, computer game centres, sports clubs, and mosques in different neighbourhoods (poor, middle class, and rich). The research was fully described to the participants in public places, and the researcher provided the adolescents with a contact number and asked them to contact the researcher if they decided to participate in the study.

Then, after explaining the study objectives and obtaining written informed consent, those who were willing to participate in the study were selected. Appointments were set with the participants and they were asked to attend to the meeting room in the Management and Medical Informatics Faculty in Kerman University of Medical Sciences, where interviews were conducted individually.

Based on the ethics fundamental regulations in medical science research in Iran, informed consent should be obtained only from the participants if they are 15 years old or over. Thus, in this study, informed consent was obtained only from the participants. A total of 18 females and 18 males were enrolled in the study. The females aged between 15 and 19 and the males between 16 and 19 years. Also, 26 participants were high school students, and 10 participants were university freshmen. All participants lived in Kerman and with their parents. None of the participants had jobs. Also, 6 participants stated that they were religious.

### Data collection

The information gathering method was the in-depth interview using semi-structured questions. The interviews were conducted by a member of the research team (N.O). On the 33rd interview, the data were saturated, but three more interviews were conducted for more certainty. After granting the permission of participants, the interviews were recorded by a voice recorder. The interviews were conducted in Persian and each lasted 15 to 50 min.

### Data analysis

One of the authors listened to the audio files of the interviews repeatedly (NO). Verbatim transcription was performed by one of the authors (NO). Two authors (e.g., NO and VYF) read implemented interviews for many times, and encoded the implemented interviews and searched the data for frequently repeated themes [25]. Then, two authors look over the codes, identified the patterns, and developed the themes. MAXQDA-V10 software was used to encode the data. The data were analysed inductively. Two other authors (MHM and SM) examined the emerging themes and ambiguities to ensure the consistency and validity of the interpretations. Next, the extracted themes were reviewed by another author, disagreements were discussed during a meeting with all members of the research team, and the themes were finalized. Finally, sustained themes were identified using constant comparisons and analytic induction. To ensure transferability, an attempt was made to use a sample of adolescents with the highest variation. Also authors attempted to present in-depth descriptions of the study method and findings. For the credibility and confirmability assurance, the copied text and its analysis were submitted to the research team and their corrective recommendations and comments were applied. Also researcher triangulation technique was used, and researchers had prolonged engagement with data.

### Ethical considerations

This study was approved by the Ethics Committee of Kerman University of Medical Sciences. The participants were assured about the confidentiality of their information and the objectives of interviewers, which were for the academic purposes. They were also informed that taking part in the study is voluntary and they could leave the interview at any time without any reason. A written informed consent was obtained from the participants and the purpose of study was fully explained to them. The interviewees were also assured that the sound recording will be stopped at any time. The interviews were initially implemented, and each interview was given an identification code to ensure confidentiality. Also, to ensure data confidentiality, all computers were secured with passwords.

## Results

The participants aged between 15 and 19 years. According to the findings of the present study, virginity had different concepts for the participants. The concept of virginity stated by the teenagers were classified into three main themes (Table 1): (1) nonacceptance of physical intimacy, with two subthemes of lack of any emotional relationship with the opposite sex and lack of physical contact; (2) acceptance of physical intimacy, with four subthemes of none penetrative relationship, virginity as a myth, virginity as a commitment, having an intact hymen, and (3) not knowing the meaning of virginity. All the interviewees, except for two females, believed that virginity is something that concerns women and not men.

**Table 1:** Findings of the Study

Main themes	Subthemes	Number of Participants	
Nonacceptance of physical intimacy	Lack of any emotional relationship with the opposite sex	Two female interviewees	“*Being a virgin means having no emotional connection with any man, except one’s husband.*” “*According to Islamic teachings, an emotional connection with any man other than one’s husband is not acceptable.*”
	Lack of physical contact	Five interviewees (four females and one male)	“*I think it’s okay to love a woman even if you text or call her but you shouldn’t have physical contact.*” *“An emotional relationship is ok as long as the man and woman don’t touch each other and only call or talk to one another.*”
Acceptance of physical intimacy	Nonpenetrative relationship	Four participants (two females and two males)	“*…..It means not having sex…..*” “*I am still virgin when I flirt but I should not have sex.*” “*Do you think kissing makes you not a virgin anymore? Not until you have sex.”*
	Virginity as a myth	Three of the female interviewees	“The concept of *virginity is ridiculous. What wise person would believe this?*” “*It’s a lie. It wants to say to women that they are not equal to men. It deprives free sexual relationship, while it is an absolute right.*”
	Virginity as a commitment	Five interviewees (three males and two females)	“*…. Virginity means loyalty to your boyfriend.*’ ‘*It means only being with your girlfriend.*” “*Virginity means being faithful to your sexual partner. It could be your boyfriend or husband. There is no difference between a boyfriend or a husband.*”
	Having an intact hymen	Fourteen of the interviewees (nine females and five males)	*“….. Not having vaginal sex”* *“For example, I can have anal sex so that my virginity is maintained.”* *“….it means having a hymen….”* *“It does not mean having no sex. It means having a hymen, which means you can have other types of sex.*”
Not knowing the meaning of virginity		Three of the interviewees (Three females)	

### Nonacceptance of physical intimacy

The first main theme was nonacceptance of physical intimacy. The theme means that from the perspective of some participants (*N* = 7), virginity means no physical intimacy, including close friendship, romantic love, or sexual attraction. This theme has two subthemes: lack of any emotional relationship with the opposite sex and lack of physical contact.

### Lack of any emotional relationship with the opposite sex

The first identified subtheme in this study was the lack of any emotional relationship with the opposite sex. This code was mentioned only by two female interviewees. The interviewees perceived virginity as lack of any emotional relationship with the opposite sex. They believed that having an emotional relationship with the opposite sex outside marriage is contrary to Islamic teachings and a good Muslim should respect the Islamic laws. Moreover, they believed that the concept of virginity is not only for women, and from the perspective of Islam, having an emotional attachment to the opposite sex outside marriage is taboo for both men and women. For example, the interviewee number 2 (a female) stated, “*We are either a Muslim or not. If we are, we should do whatever our religion tells us to do. If our religion says a man and a woman should have no emotional relationship outside marriage, we must accept it. As a Muslim, if I have a relationship with the opposite sex even via phone, I am not a virgin.*”

### Lack of physical contact

The second identified subtheme in this study was the lack of physical contact. Moreover, five interviewees (four females and one male) believed that virginity means lack of physical contact between a woman and a man before marriage, which includes pats on the back, touches on the arm, shoulder, or face, hugs, and holding hands. Having an emotional relationship with the opposite sex via phone, chat, and even going on dates is acceptable and even necessary if there is no physical contact. They considered these relationships necessary for getting to know their potential spouses. However, having physical contact was not acceptable to them and they considered a sin. The interviewee number 5 (a female) said, “*Without communicating with the opposite sex, it is impossible to make a decision for marriage, but you have to control yourself. I mean, if I have a date with a man, I’m still a virgin, but I should not have physical contact with him because it is a sin based on Islamic teachings.”* This narrative points out that adolescents have different understandings of Islamic teachings about relationships with the opposite sex and committing a sin.

### Acceptance of physical intimacy

The second main theme was acceptance of physical intimacy. The theme means that from the perspective of some participants (*N* = 26), virginity means a range of physical intimacy that includes sexual activity. However, this range was different among the participants. This theme contains four subthemes: nonpenetrative relationship, virginity as a myth, virginity as a commitment, and having an intact hymen.

### Nonepenetrative relationship

The first identified subtheme was nonpenetrative relationship. Four participants (two females and two males) believed that emotional relationship between a man and a woman is inevitable, and physical intimacy such as hugging and kissing occurs in such relationships. However, what threatens the concept of virginity is intercourse.

Interviewee number 10 (a female) stated, “*Nowadays, everyone has boyfriends. Well, both sides have certain needs, but women should be more careful since men are not at risk of losing their virginity.”* The interviewee number 12 (a male) also said that, “*As a man, unlike my dad, I do not think that a virgin woman is someone who has never had a relationship with anyone. Such a thing is impossible these days. I personally think that it is not a problem if she has had a boyfriend, but it is important to me that she had no sexual relationship.”*

The interviewees considered virginity as a female-related issue. Unlike their parents, they did not think that having an emotional relationship with the opposite sex should be avoided until marriage. Rather, they defined a range for such a relationship and believed that this type of relationship is acceptable if there is no sexual intimacy.

### Virginity as a myth

The second identified subtheme was virginity as a myth. Three of the female interviewees considered virginity as a concept that is completely related to the female gender. To them, virginity was meaningless and a myth. They perceived it as a fictional story that has become a custom over time and refers to the inequality between women and men, exploitation of women by men, and a bitter myth. Virginity had no meaning or value to this group of interviewees. The interviewee number 14 (a female) said, “*A woman is a free human being. Virginity is a lie that men have made up and repeated over time to make women believe it. Why they are not virgins themselves, and what does it mean to be a virgin anyway?*”

Interviewee number 30 (a female) stated, “*…This is my body. I have to decide for it, not men. Virginity is a myth and a clear indication of inequality between men and women.”*

### Virginity as a commitment

The next identified subtheme in this study was virginity as a commitment. Five interviewees (three males and two females) believed that virginity means two people’ commitment in a relationship. These two individuals might have had relationships with others in the past, but this does not mean the loss of virginity. Virginity means being committed to your emotional partner in a relationship. Therefore, a person’s virginity is revealed over time in a relationship. Interviewee number 33 (a male) said, “*Having no relationship does not mean being a virgin. Virginity means being just with your partner in a relationship. I don’t care about the past life of my girlfriend, even my wife. However, from the day she becomes my wife or girlfriend, it will be important to me if she keeps her virginity. If she does not cheat on me when she is with me. Her past does not matter to me*.”

Interviewee number 19 (a female) stated, *“…. I can give you a lot of examples of those who had a hymen, but cheated on their husbands after marriage. Being a virgin means being committed to your husband.”*

For some interviewees (*N* = 5), it seemed that the meaning of virginity was different from the meaning of having a hymen. They regarded virginity as being committed and faithful. In fact, having a hymen was not symbol of virginity for them.

### Virginity as having an intact hymen

Virginity as having an intact hymen was another subtheme identified in this study. According to 14 of the interviewees (nine females and five males), virginity meant having an intact hymen. They believed that virginity does not necessarily mean lack of sexual intercourse, but having an intact hymen. Thus, in their views, sexual relations such as anal sex and genital touching were acceptable, and having these types of relations were not considered as violation of virginity.

Interviewee number 20 stated, *‘Virginity means having a hymen. When a woman has sex on her wedding night, if no vaginal bleeding occurs, it will be a disgrace for her and it means she had sex prior to her marriage. However, we can have anal sex before we get married, and nobody will find out about it.”*

Fourteen participants considered the meaning of virginity as having a hymen rather than chastity. It was important for them to have a hymen because of Iran social norms, but it did not mean that they did not have other forms of sexual relationship. In fact, social norms did not lead to abstinence.

### Not knowing the meaning of virginity

The last main theme identified in this study was not knowing the meaning of virginity. Three of the interviewees did not know the meaning of the term ‘virginity’.

## Discussion

The findings of the present study varied from the nonacceptance of any relationship to the acceptance of vaginal intercourse. Generally, all of these concepts are categorised into two main themes, including nonacceptance and acceptance of physical intimacy.

The main theme of nonacceptance of physical intimacy was the subtheme of lack of physical contact. It indicated that the relationship with the opposite sex in the form of social networking, chat, phone call, and going on dates without physical contact was acceptable for the participants. Only one male mentioned this subtheme. This may mean that this meaning of virginity was less accepted in males than in females. Various studies showed that the likelihood of a romantic date leading to sexual intercourse is considerably high [26].

Also, the main theme of nonacceptance of physical intimacy contained the subtheme of lack of any emotional relationship with the opposite sex. According to this view, the concept of ‘virginity’ means the absence of any emotional relationship with the opposite sex outside the framework of marriage, and this concept is not specific to the female gender. This view is completely consistent with the policymaking approach used in the field of premarital sexual relations in Iran. However, it should be noted that only two female interviewees had such an opinion. This may indicate that the meaning of virginity as lack of any emotional relationship with the opposite sex was more likely to be accepted by females than by males, although only two out of 18 females mentioned it. Motamedi et al. showed that very few participants agreed with the lack of premarital emotional relationship with the opposite sex [27] before marriage. Therefore, it seems that Iranian health policymakers have not been able to integrate the concept of virginity as a value into the value system of adolescents.

Acceptance of physical intimacy was another main theme which was the most frequently mentioned theme in this study. The most frequent concept that the interviewees referred to was ‘virginity as having an intact hymen’. Also, 9 female participants and 5 male participants mentioned this subtheme. This may indicate that the meaning of virginity as having an intact hymen was more likely to be accepted in both genders, especially in females. Females emphasized the importance of having a hymen more, which may be due to the fact that having an intact hymen for a female is highly important in the Iranian society and lack of it has serious consequences. This theme implies that any form of sexual intercourse with the opposite sex in the absence of vaginal intercourse is acceptable and does not harm the concept of virginity. In fact, interviewees linked the concept of virginity as a social concept to having a hymen as a physiological topic. Moreover, 14 participants perceived virginity as having an intact hymen and believed that virginity was only particular to girls and not to boys. Ahmadi referred to the relationship between the concept of virginity and having an intact hymen in the context of Iran and believed that the relationship between these two concepts is one of the reasons for increasing the demand for hymenoplasty [16].

The main theme of ‘acceptance of physical intimacy’ included other subthemes such as nonpenetrative relationship, virginity as a myth, and virginity as a commitment rather than just not having a relationship. These themes are new interpretations of virginity that provide a different concept for virginity as ‘not touched’. The interviewees, who considered virginity as a myth, perceived this phenomenon as a male-dominated view toward the female body which belongs to the distant past. This concept was mentioned by 3 female participants and not mentioned by any male participants, which may indicate that the meaning of virginity as a myth was more likely to be accepted by females. The approach is influenced by the feminist view on the concept of virginity. Milles et al. referred to the role of feminist approach in altering the concept of virginity [28]. Also, 3 male participants and 2 female participants mentioned virginity as a commitment. The interviewees who defined virginity as a commitment or a myth had a different interpretation, far from the current context and condition of Iran. In fact, such interpretation reflects a change in the value system of interviewees and the breakdown of social taboo on the lack of hymen, which is the greatest sign of virginity in the Iranian culture. Motamedi et al. referred to the change of value system of participants regarding premarital sex [28].

Another subtheme derived from the main theme ‘acceptance of physical intimacy’ was nonpenetrative relationship. This concept was mentioned by 2 male and 2 female participants. According to the participants, any other sexual relations, except for penetrative ones, are accepted and do not harm the concept of virginity. The difference between this subtheme and the subtheme of virginity as having an intact hymen is in accepting anal intercourse. Both of these themes are the results of dominant approach in Iranian society regarding virginity and its relationship with an intact hymen for females. These findings suggest that the probability of getting sexually transmitted disease is high in other types of sexual relations [29].

Although the most reliable policy on STDs prevention is based on the lack of premarital sexual intercourse, the success of this policy is related to the concept of virginity [22, 30]. Since Iran is an ideological country, and according to Islamic teaching having sex outside marriage is not accepted and is associated with legal and religious punishments, the preferred policy on premarital sexual relations is the policy of banning such relations and respecting purity. Moreover, no education on sexual relation and its risks is provided for the adolescents who are highly prone to such behaviours [31]. On the other hand, virginity is considered as a value in the Iranian culture [14]. Therefore, based on the findings of the present study, 26 of the 36 participants in this study did not consider the various forms of physical intimacy as the violation of virginity and also did not have sufficient information about STDs. This may indicate that adolescents with such attitude would probably enter in premarital sexual relations without knowing the dangers. On the other hand, since virginity is only meaningful for females according to the perspective of some interviewees, and studies confirm the existence of such view in the context of Iran [32], the policy of observing chastity, purity, and virginity until marriage cannot grantee the sexual health of male teenagers. Considering the changes in the transmission pattern of AIDS in Iran from injection with a common syringe to sexual intercourse [33], the perspective of interviewees can be a wake-up call for the health policymakers.

Our findings indicated that most interviewees associated the concept of virginity only with females and believed that virginity is meaningless to males. They believed that virginity means having an intact hymen in females [34] and the reason for such a belief is rooted in different concepts of virginity in the context of Iran. Thus, having virginity only for females in Iran has a long history and means chastity, virtue, and modesty for women [16].

## Conclusion

Iran is an ideological country where sexual relations are prohibited outside marriage framework. According to Islamic teachings, having sex outside the marriage framework is not accepted and is associated with legal and religious penalties and punishments, so the selected policies regarding the premarital sexual relations are based on the banning of such relations and respecting purity. Although the most reliable policy on the prevention of STDs is the lack of premarital sex, the success of this policy is closely related to the concept of virginity. The findings of the present study showed that from the perspective of Iranian adolescents, virginity has a wide range of concepts from denying any emotional relationship with the opposite sex to accepting physical intimacy and penetrative relationship. Most interviewees did not consider physical intimacy in any form as the breach of virginity. Thus, the policy of purity and lack of sexual relationship before marriage is inadequate and associated with the risk of STDs, according to the concept of virginity from the perspective of Iranian teenagers. Therefore, Iranian health policymakers must take steps towards modifying the concept of virginity in the value system of adolescents and provide sex education courses to this population group.

## Data Availability

The datasets used in this study are available upon reasonable request.
